# A Case of Polypoidal Choroidal Vasculopathy Associated With Optic Disc Drusen

**DOI:** 10.7759/cureus.10297

**Published:** 2020-09-07

**Authors:** Krishnan Sarojini, Kiet Phang Ling, Wee Min Teh, Haslina Ali, Embong Zunaina

**Affiliations:** 1 Department of Ophthalmology, School of Medical Sciences, Universiti Sains Malaysia, Kubang Kerian, MYS; 2 Department of Ophthalmology, Hospital Sultanah Bahiyah, Alor Setar, MYS; 3 Department of Ophthalmology, Hospital Universiti Sains Malaysia, Kubang Kerian, MYS

**Keywords:** optic disc drusen, polypoidal choroidal vasculopathy, choroidal neovascularization

## Abstract

We report a case of optic disc drusen (ODD) associated with peripapillary polypoidal choroidal vasculopathy (PCV). A 62-year-old Malay lady presented with both eye ODD and the left eye associated with peripapillary subretinal hemorrhage. Ultrasound B-scan and red-free photography confirmed the optic nerve head drusen findings bilaterally. Optical coherence tomography (OCT) of the left eye showed sharply elevated peripapillary pigment epithelial detachment with subretinal fluid. The presence of peripapillary polyps with branching vascular network in indocyanine green angiography of the left eye further confirmed the diagnosis of PCV and excluded choroidal neovascularization (CNV) secondary to ODD. Subsequently, the patient was treated with a combination of verteporfin photodynamic therapy with three monthly intravitreal ranibizumab injections. Three months after the combined treatment, OCT showed completely resolved subretinal fluid. ODD can cause compression of the subretinal vessels at the optic disc that results in retinal ischemia and release of vascular endothelial growth factor, which may trigger the development of CNV or PCV. The rarity of this combination makes it interesting to study more cases of ODD with PCV. Importantly, a thorough evaluation in distinguishing the PCV from the CNV that mimics it is crucial for early detection and prompt intervention. In this case, indocyanine green angiography (ICGA) is the diagnostic method to differentiate the PCV from CNV secondary to ODD.

## Introduction

Optic disc drusens (ODD) are deposits, usually calcified hyaline-like deposits, within the substance of the optic nerve head. It presents in up to 2% of the population and often bilaterally (75%). It is an autosomal dominant inheritance [1,2]. Peripapillary subretinal hemorrhage is a rare complication of ODD as a result of direct mechanical compression and rupture of subretinal vessels at the optic disc [2]. Mechanical compression can also cause impairment of peripapillary circulation that leads to retinal ischemia and trigger the development of choroidal neovascularization (CNV) or polypoidal choroidal vasculopathy (PCV) [3].

PCV is an inner choroidal abnormality characterized by polypoid dilatations of choroidal vessels with branching vascular networks. It is known as a subtype of neovascular age-related macular degeneration because it resembles similar morphological features [4]. To best of our knowledge, PCV associated with ODD has not been reported. Here we report a case of peripapillary PCV associated with ODD.

## Case presentation

**Figure 1:**
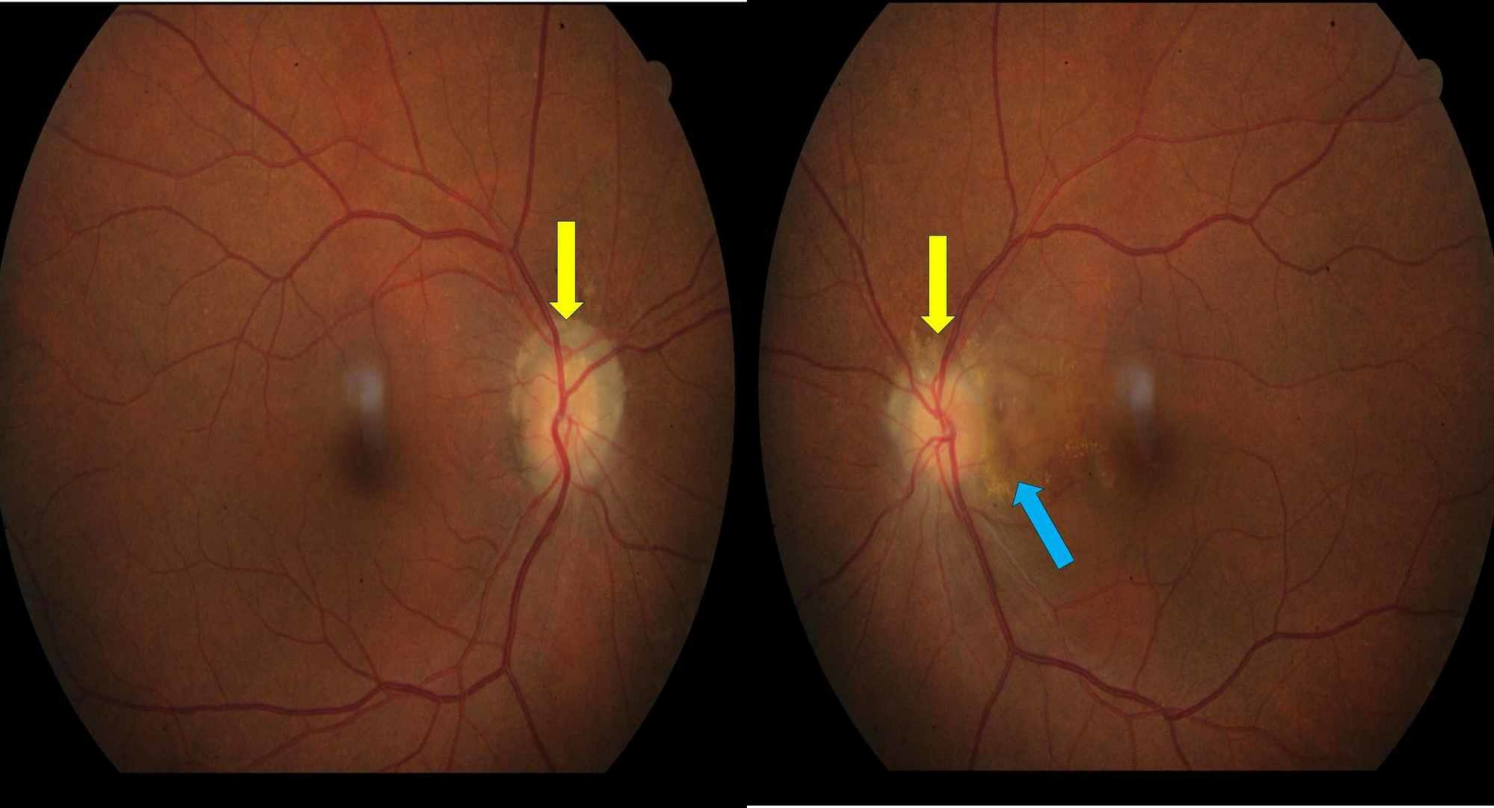
Color fundus photo of both eyes with indistinct and irregular disc margin, with “lumpy-bumpy” appearance (yellow arrow) and presence of orange nodular lesion temporal to left optic disc with subretinal hemorrhage (blue arrow).

A 62-year-old Malay lady with no known comorbid presented to the ophthalmology department with left eye central scotoma for a month duration. Otherwise, there was no other significant associated symptom. The visual acuity was 6/24 in the left eye and 6/9 in the right eye with intraocular pressure of 16 mmHg bilaterally. There was no relative afferent pupillary defect. Anterior segment revealed unremarkable findings with immature cataract bilaterally. Posterior segment examination showed both eyes ODD with “lumpy-bumpy” appearance and indistinct irregular disc margin. In addition, the left eye ODD was associated with an orange nodule temporal to optic disc with minimal subretinal hemorrhage (Figure 1) (Abstract of unpublished E-poster: Krishnan S, Ali H, Ling KP, and Teh WM. Is Optic Disc Drusen Associated with Idiopathic Polypoidal Choroidal Vasculopathy? 11th Asia-Pacific Vitreo-Retina Society Congress (APVRS) 2017, Kuala Lumpur Convention Center, Malysia; December 8-10, 2017).

**Figure 2:**
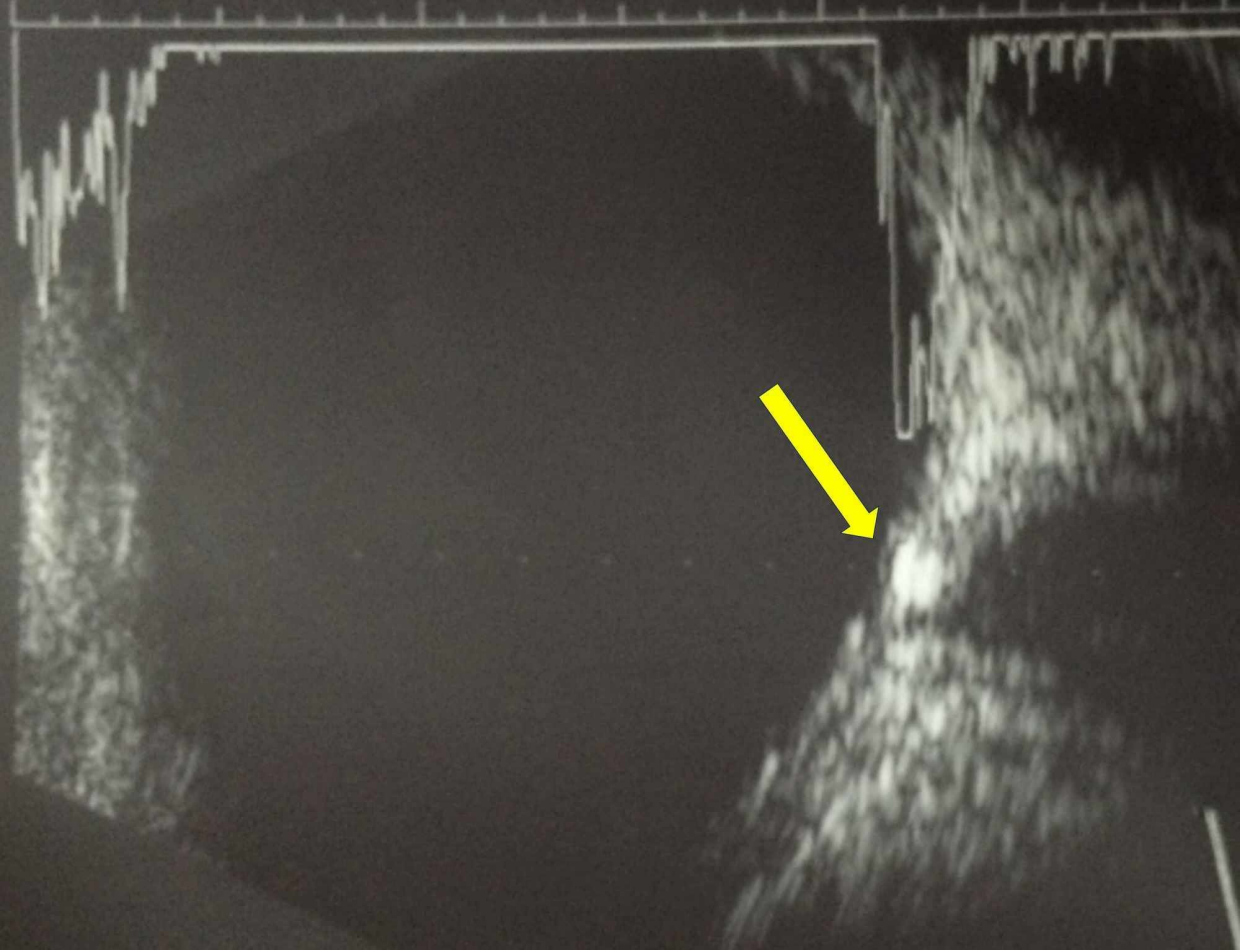
Ultrasound (B scan) shows highly reflective oval lesions at the right optic disc (yellow arrow), which represent the calcification in optic disc drusen.

**Figure 3:**
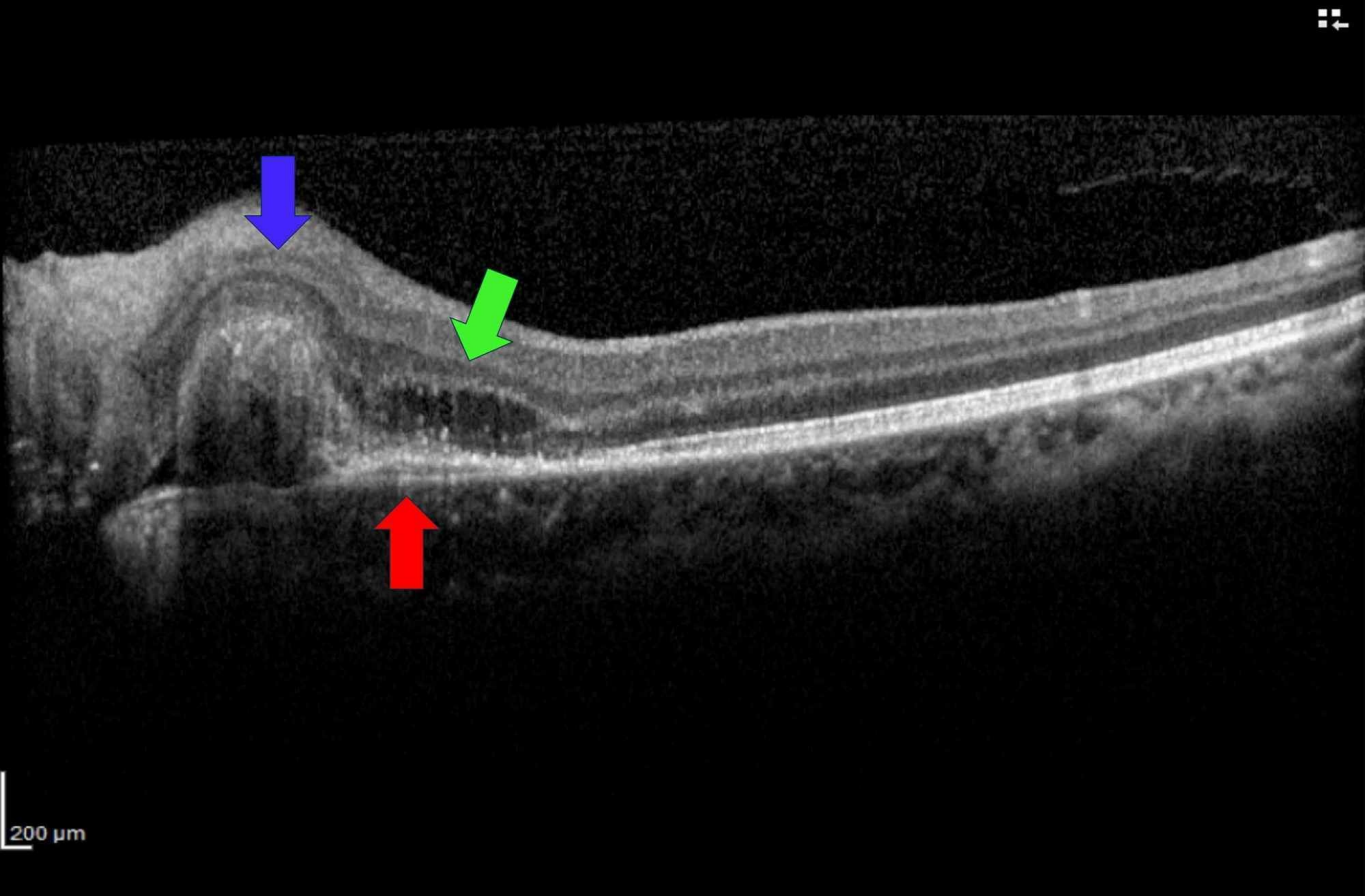
Optical coherence tomography (OCT) of the left eye shows peripapillary pigment epithelial detachment with heterogeneous hyperreflectivity, which indicates the presence of polyps (blue arrow) with subretinal fluid (green arrow) and double layer sign (red arrow).

**Figure 4:**
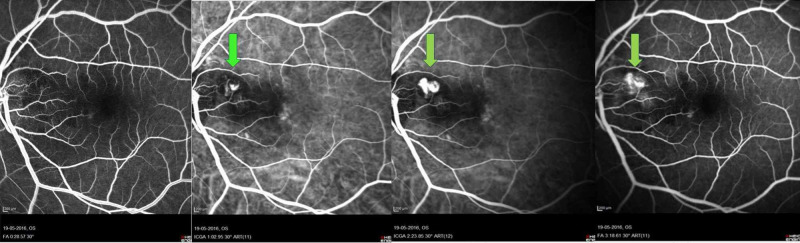
Indocyanine green angiographies (ICGA) of the left eye showing polypoidal lesion with branching vascular network (green arrow).

Ultrasound B-scan showed moderately high reflectivity over the elevated optic disc consistent with surface ODD (Figure 2). Red-free photography revealed autofluorescence of the drusen bilaterally without fluorescein administration. Optical coherence tomography (OCT) macula of the left eye showed peripapillary pigment epithelial detachment at the region corresponding to the nodule, with subretinal fluid approaching the fovea level (Figure 3). The peripapillary pigment epithelial detachment was associated with a double-layer sign suggestive of PCV. Indocyanine green angiographies (ICGA) of the left eye revealed the presence of peripapillary polyps with branching vascular network that further confirmed the diagnosis of PCV and excluded CNV secondary to ODD (Figure 4) (Abstract of unpublished E-poster: Krishnan S, 2017).

Thus, her left eye was treated with combination therapy, including a photodynamic therapy (PDT) session, followed by three monthly intravitreal anti-vascular endothelial growth factors (VEGF) injection. After three months of injection, OCT showed completely resolved subretinal fluid and her left eye vision was improved to 6/9. During 12-month follow-up period, no recurrence was noted and visual acuity remained stable (Abstract of unpublished E-poster: Krishnan S, 2017).

## Discussion

ODD are benign acellular deposits resulting from calcific degeneration and deposition of calcium, amino acids, ribonucleic acids, and iron anterior to the lamina cribrosa. The course of the disease usually occurs early in life and may progress, in which ODD becomes more visible in the first two to three decades [1,2]. Almost 60% of ODD have visible drusen on optic nerve head, with clinical features of refractile, pale, rounded, and vary in size from tiny foci to large lobulated clusters. On the other hand, buried ODD has more various clinical features from normal disc appearance to mimicking a swollen disc, often misdiagnosed as papilledema [5].

Histology of ODD shows acellular, noncapsulated, with concentric laminations. The surrounding area shows the presence of localized atrophy, occasionally associated with overlying neuroretinal gliosis. Besides that, there was the presence of cystoid bodies that represents the neurodegenerative structures in the deep peripapillary retina [1,2].

Most of the patients are asymptomatic and generally stable. It is often an incidental finding upon routine fundus examination. However, mild visual symptoms, visual field defects, and rarer complications have been reported as well. The superficial ODD is diagnosed clinically, whereas buried ODD is usually required additional diagnostic testing, such as ultrasound, fundus fluorescence angiography, optic disc autofluorescence, and OCT [5]. In addition, investigation is also needed to detect possible complications. Reported complications include enlargement of the blind spot, optic disc hemorrhage (2%-10%), vitreous hemorrhage, CNV, PCV, serous maculopathy, and nonarteritic anterior ischemic optic neuropathy (NAION) [2].

PCV resembles multiple serosanguineous bilateral retinal pigment epithelial detachments. The presence of prominent hemorrhage should lead to the consideration of PCV, particularly if there is an absence of drusen and the patient is relatively young and Asian or black. ICGA is essential for the definitive diagnosis of PCV [4,6,7].

The possible mechanisms for ODD complications were postulated due to compressive effect by the ODD at surrounding subretinal blood vessels, which caused disruption and congestion of vascular integrity, subsequently leading to retinal ischemia. This process induces the release of VEGF, which may trigger the development of CNV or PCV [1,2,8]. The pathogenesis of PCV also had been explained due to disruption in choroidal circulations, which was evidenced by the pachychoroid. A study based on biomicroscopy of PCV supported the similar mechanism of compression effect from a sclerosed vessel in branch retinal vein occlusion (BRVO), causing vascular fragility, degeneration, polypoidal formation, vascular leakage, and hemorrhage [9]. In addition, the presence of polyps in the early phase and leakage in the late phase in an ICGA further enhanced this theory [4,9].

Nakano et al. reported an association between PCV and optic disc coloboma. The proposed pathogenesis was that abrupt termination and abnormality in the retinal pigment epithelium and Bruch’s membrane architecture in a retinochoroidal coloboma may lead to subretinal neovascularization [10,11]. There was another report that showed the presence of PCV at the end of peripapillary choroidal cavitation (PCC) in optic disc coloboma [12]. The development of PCV in association with choroidal cavitation speculated may be due to its abnormal choroidal morphologic changes at the end of PCC that lead to mechanical stress and trigger VEGF production [10]. Furthermore, 20%-30% of the eye has cilioretinal branches from choroidal origin. The ODD can cause the obstruction which may lead to PCV like CNV features and cause significant visual distortion. In our case, a similar mechanism might occur due to enlarging of ODD, inducing a compression effect at the peripapillary region, which causes abnormal choroidal morphological changes and VEGF production, and subsequently a PCV development.

The prevalence of ODD is very low and their association with CNV and PCV is extremely rare, with no established treatment regimen [4]. The management of PCV varies according to the clinical-pathological manifestations of the disease. It includes monotherapy with intravitreal anti-VEGF, verteporfin PDT, and combined therapy with anti-VEGF and PDT [7]. Combination therapy showed encouraging results based on complete polyp regression and also in terms of achieving best-corrected visual acuity compared to anti-VEGF monotherapy [6]. In our case, the combination therapy showed an excellent outcome.

## Conclusions

This is the first case report describing an association between PCV and ODD. The rarity of this combination makes it interesting to study more cases of PCV and ODD. The morphological changes due to enlarging ODD may cause the development of PCV in this case. Importantly, PCV presenting with ODD must be distinguished from CNV that mimics it as the early detection and appropriate intervention is crucial. Furthermore, the management is different in both entities. In our case, combination therapy of PDT and anti-VEGF injection was effective.
